# Social Implications of Weight Bias Internalisation: Parents’ Ultimate Responsibility as Consent, Social Division and Resistance

**DOI:** 10.3389/fpsyg.2019.02321

**Published:** 2019-11-21

**Authors:** Sharon Noonan-Gunning

**Affiliations:** Department of Sociology, City, University of London, London, United Kingdom

**Keywords:** parents, stigma, care, responsibility, class, inequalities, moral associates

## Abstract

Responsibility is a moral quality of caring that is central to child health policies. In contemporary United Kingdom these policies are based on behavioural psychology and underpinned by individualism, an ideology central to neoliberal governance. Amid the complexities of “obesity” and inequalities, there is a multi-layered stigmatisation of parents as moral associates. Few studies consider the lived realities of food policy processes from the standpoint of class. This critical qualitative research draws on theorists who explain processes of power and class: Foucault, Gramsci, Bourdieu, and Marx. Its objectives are: (a) to understand the lived experience of parents as they interact with food policy; (b) to explore how parents resist stigmatisation; and (c) to reflect on implications for policy and practice.

**Methods:**

Using purposive sampling, 31 ethnographically informed interviews were carried out in a London borough, with policy actors: policymakers, implementers, and parents as policy recipients, including 12 working-class mothers.

**Results:**

A core theme of “responsibilities” emerged with four interconnecting sub-processes that provide insight into how stigmatisation and resistance evolve through policy.

**Discussion:**

As have others, this study reveals the idea of responsibility as fundamental to the processes of soft power. Child health is a priority for participants and a “ruling idea.” The diffusion of responsibility throughout policy leads to confusion about where it lies. New subjectivities are formed in line with ideas of governmentality. Parents engage with policy at multiple sites that elicit symbolic violence, and stigma sows social divisions. Against this background, working-class parents are left in a state of cognitive dissonance between being made responsible (responsibilisation), and feeling responsible (self-blaming) for their children’s weight while lacking the material resources to provide an optimal nutritious diet. Resistance is interwoven and is essentially found in parents’ policy alternatives that diverge from United Kingdom government policy.

**Conclusion:**

Critical qualitative research using multiple theorists is valuable in understanding how parents interact with policy in a complex social world. With United Kingdom policy failing, useful insights are provided into how involving parents in policymaking might determine a meaningful collective responsibility, with a political ethic of care and unity between parents that would advance health equity.

## Introduction

Children’s rights are emphasised in food and health policies. Yet in practice their rights to be free from hunger and poor health are disregarded by United Kingdom governments (Booth, 2019). “Child obesity”^1^ is situated in this contradictory context. On the one hand it is the greatest threat to the future of children, a societal burden with huge health and economic costs, and cause of inequalities (Department of Health, 2018). On the other, policies continually fail, resulting in intractable prevalence rates that are accompanied by a deepening social gradient (House of Commons Health Committee, 2018). Such contradictions can be understood in the context of contemporary neoliberal society – a political economic system underpinned by the belief that human advancement is best served by a free market economy and ideology based on individual freedoms, rights and responsibilities. The focus on the individual is central to health psychology, a discipline that together with behavioural sciences has become integral to public policies (Jones et al., 2013). So, “child obesity” policies focus on changing individual parents’ food practices rather than addressing the structural influences contained within the physical and social environment.

Neoliberal societies are found to be highly unequal (Schrecker and Bambra, 2015). Inequality and stigma are linked because stigma as a social process plays a key role in producing power relations, social control, and in the devaluation, discrimination and exclusion of specific groups; processes that support the dominant social order (Parker and Aggleton, 2003, p. 16). There is a belief among wealthy elites that inequality benefits their social power; consequently, fear is created, shifting blame to “others” in a “political need to blame the poor” (Dorling, 2018, p. 18). Blame and its internalisation mask the structural factors responsible for the inequalities and injustices generated by neoliberal policies are concealed. Stigma is used to motivate behavioural change (Pont et al., 2017). It is “weaponised” by the state (Scambler, 2018) or emerges as an unintended consequence of policies that, for example, use fear-based messaging (O’Hara, 2014). It also emerges in “othering” processes such as health surveillance programmes that measure and differentiate children as “healthy weight” or obese (Nnyanzi et al., 2016); programmes aimed at identifying population trends, not individual clinical diagnosis (Dinsdale and Ridler, 2010).

Whatever its driving force, the generation of blame and self-blame has harmful effects for children and young people (Pont et al., 2017). The United Kingdom has experienced an increase in stigmatisation and shaming discourses (Bissell et al., 2016; Tyler and Slater, 2018). Weight bias is pervasive and multilayered (Puhl and Latner, 2007; Bresnahan and Jie, 2016), with a greater impact on working-class parents as suggested by the material differences that underpin the social gradient. While the lived effects of weight bias are considered, few researchers explore its intersect with class (Bissell et al., 2016; Zivkovic et al., 2018). Health indicators, such as the social gradient, do not reflect the lived experience of class (Navarro, 2009).

The growth of stigmatisation in the United Kingdom provides a context in which obesity has taken on meaning beyond clinical diagnosis. Obesity is highly stigmatised and includes parents as moral associates, because as primary caregivers they are given core responsibility for their child’s weight: “in the West, children’s large bodies have become visible markers of parental irresponsibility” (Davis et al., 2018, p. 61).

### The Concept of Responsibility in Neoliberalism

Responsibility is a not a straightforward or abstract concept; rather, it is a process involving social relations underpinned by ideology and the material needs around caring for children. It concerns societal ethics and social cooperation in the distribution of responsibilities and care. These are political decisions (Williams, 2005; Tronto, 2013). Neoliberal ethics are based on individualism and rational choice in which only personal responsibility matters. So, tensions would be expected with policies that frame responsibility as “collective” or “shared” between the state, the food industry and parents, as it becomes unclear who has and who escapes the burden of caring responsibilities (Tronto, 2013, p. 60; Gillies et al., 2017, p. 67). There is ambiguity about moral responsibility and who has power to take action to protect child health.

In the neoliberal economy, the state functions to maintain the free market as part of a political-economic project, characterised by privatisation, deregulation and a low-wage economy (Tronto, 2013, p. 38). Working conditions are precarious and real wages have not increased since 2010 in the United Kingdom (Collinson, 2019). The state is restructured and decentred, so that it operates through multiple sites, including the local state, and through collaboration between actors with different interests. However, although hollowed out, it retains an overarching power (Gillies et al., 2017, pp. 66–69). For Jones et al. (2013) it is a “psychological state” that adopts behavioural economics.

Behavioural power is exercised through the concept of responsibility (Peeters, 2019) which shifts between social actors, usually from state agencies onto others (O’Malley, 2008). For example, Ditlevsen et al. (2016) found that responsibility shifts from health professionals onto families. These are not inert processes. They create new subjectivities for policy actors (Monaghan et al., 2010). The state is involved in this subject formation by creating a parent-self who fulfills neoliberal policy requirements (e.g., Gillies, 2011). This competency-based parent, self-governs and socialises children; regulating, monitoring and disciplining to enable healthy choices and bodies, and responsible consumption (Gillies, 2011). But tensions arise based on class, cultural and socioeconomic differences with affordability as a core question. Despite this, parents in poverty are highly resourceful (Caraher, 2016) and juggle caregiving commitments, that Davis et al. (2018) considers to be morality work in which they navigate multiple moral burdens and utilise multiple strategies based upon their own experiences. These lived experiences are lacking in obesity research.

### Food Industry and Responsibility

While parents face public scrutiny and sanctions that ultimately can involve child safeguarding, policy only requires voluntarism on the part of the food industry, which is given power in public health policymaking through partnership-working with government (Department of Health, 2011). These tensions are compounded by “irresponsible” market processes that operate in the background as part of political, economic and cultural decision-making (Tronto, 2013, p. 60). For example, choice has flourished, with 20,000 new food products every year (USDA ERS, 2013 cited in Lang and Heaseman, 2015, p. 16), they are “edited” and constructed by advertising, and tracked and targetted by algorithms (Lang and Heaseman, 2015; Mahoney, 2015). Similarly, the needs of the market rather than those of community drive the spatial planning of the food environment; a responsibility of local government. Correlations are found between market liberalisation and increases in fast-food consumption (Winson, 2014; Otero et al., 2015), and mean body mass index (BMI) (De Vogli et al., 2014). Obesity in children in the United Kingdom is correlated with the density of fast-food outlets as well as deprivation (National Obesity Observatory, 2012).

### Resistance

Resistance is inherent to social inequalities and stigmatisation. For the individual this can mean the “capacity to resist, counteract or otherwise remain unaffected” by the stigmatisation (Lau et al., 2017, p. 72). It involves stigmatised people distancing themselves from stigmatising labels by negotiating alternative social meanings (De Brun et al., 2014). It takes the form of symbolic protest, refusal to comply with policies, and reframing of moral meanings (Warin et al., 2008; Zivkovic et al., 2018). Parker and Aggleton (2003) focus on how people respond as communities of resistance that challenge stigmatisation and its internalisation.

In relation to food and parents, stigma continually evolves and is amplified, even in resistance. Stigma is attached to working-class foods, as exemplified by the ‘Battle of Rawmarsh’ (Wainwright, 2006). In Rawmarsh, a United Kingdom working-class community, school menus were changed based on the healthy-eating recommendations of a celebrity chef, but without consultation with parents. Mothers took chips to school to ensure their children ate familiar foods but the national media response was to vilify them as “sinner ladies” (Fox and Smith, 2011). The story illustrates how parents’ engagement can produce solutions based on resourcefulness and experience, as well as how resistance can amplify stigmatisation.

This article draws on doctoral research that explored disconnects between parents’ social reality and food policy. Stigma was not looked for, but it cut through parents’ lives. Its counter-productivity that was in my study is recognised by public health policy thinkers, who called for change “to end the blame game” and for a shift to empathy and support (Hochalf et al., 2019). However, such efforts do not change the trajectory of policy away from individual responsibility and thus would not counter stigma. In contrast, in my research parents’ experiences and policy solutions provide insight into a collective community ethic of responsibility and care for children.

This study aims to contribute to transdisciplinary and critical communities within health psychology, dietetics and policy studies. There is little research on parents as moral associates, on their lived experiences, or as a community of resistance that advances policy change to benefit children’s health. If policy is to tackle the potentially harmful effects of stigma, it needs to reach beyond psychological and behavioural perspectives to explore structural social relations (Tyler and Slater, 2018). Arguments for participatory health equity in all policies are relevant (O’Keefe, 2000), with this requiring the meaningful involvement of parents in policymaking and a greater reflexivity among policy implementers about our roles in policy processes. Drawing on Murray (2015) this paper examines the connections between the individual, structures and power, and supports a psychology that is socially engaged and historically specific. Change is understood as constant. Ultimately, a critical psychology seeks to improve the “health of the world’s masses … in doing so, they must address the issues of power and who wields it, of powerlessness and how it is connected to ill health” (Murray, 2015, p. 9).

### Critical Research and Power

Multiple theorists are drawn on in this work to understand the complexities of social life (Parker and Aggleton, 2003; Jones et al., 2016). Power and class are considered key social factors involved with stigmatisation as a social process that intersects with policy. This aligns with a critical research approach conceptualised by Kincheloe and McLaren, which assumes “all thought is mediated by power relations that are historically and socially constructed” (Denzin and Lincoln, 2011, p. 164). The critical research agenda aims to empower. Accordingly, this study prioritises parents’ views, and how parents might be involved in policymaking. Epistemologically, critical hermeneutics explores the formation of knowledge through language, meanings and interpretations that look for power dynamics. It is relevant to exploring policy processes because “it grounds critical research that attempts to connect the everyday troubles individuals face to public issues of power, justice and democracy” (Kincheloe and McLaren, 2003, p. 449). The ontology of Marxist dialectics is relevant because its social reality is one of constant change, is relational, and maintains that change is driven by internal contradictions (Ollman, 2003).

### Critical Theories of Power and Class

**FIGURE 1:**
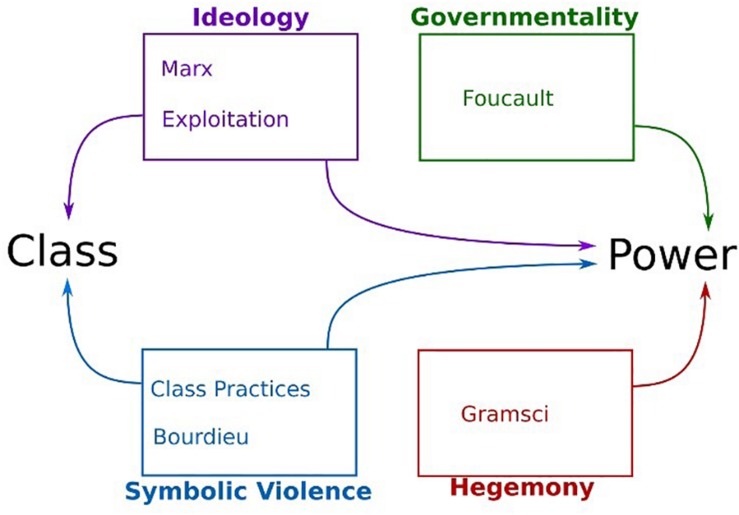
Key theorists’ contribution to exploring class and power.

This study draws on theories of soft power, that is, power wielded not through coercion but through consent: Gramscian hegemony, Foucauldian governmentality, Bourdieusian symbolic violence and Marx’s “ruling idea.” Taken together, these theories provide a powerful understanding of the complexity of the social world. Each theory challenges social oppression, and they complement each other by providing theoretical lenses through which to consider the everyday lives of parents from different viewpoints as they intersect with the policy process. As Figure 1 illustrates, Marx provides, in relation to class, a macro-level understanding of processes of exploitation that constitute classes within capitalism. This is complemented by a Bourdieusian approach to social practices, according to which social reproduction takes place through “fields” of action and access to capitals – economic, cultural/symbolic, and social – that constitute our class habitus.

In relation to power processes, complementarity is found. Marx maintained that soft power is wielded through ideology as a means of maintaining class relations, notably through “ruling ideas” that appear as common sense and are thus adopted by the working classes and their communities. Similarly, Gramsci (1971) hegemony considers how consent is negotiated and won for the ideas and values that support the dominant class. It involves the state and cultural spheres, and the latter involves civil society. In contemporary society, Gramscian theory considers how cultural life, beliefs and ideas are shaped and reproduced as hegemony, such as in universities and the media. Through the ruling idea and hegemony, a vertical view of power processes is found; this is complemented by the horizontal view of Foucauldian governmentality, which examines power at the micro-person level, and by Bourdieusian symbolic violence.

For Foucault, power is examined at the micro-person level and in institutional sites. Its processes are dynamic and relational, they circulate, and they are productive and positive as well as oppressive. Resistance is inherent and becomes productive of change. Foucault examined the historical relationships of power, such as how control is maintained by punishment or discipline. In particular, he considered how the mechanism of self-regulation and the disciplining of the self evolved through the surveillance techniques of modern prisons. His work illustrates how the processes of surveillance lead to perpetual self-surveillance and self-supervision, and to the “internalisation of the supervisor” (Foucault, 1975, p. 146).

Processes of individuation and normalisation work through “various examinations” and assessments, with relevant examples being BMI, including that of children. In assessing and recording the individual, individuation is produced, which reinforces the notion of social division and individual differences, particularly between those who do and those who do not conform (the “other”). The focus on the power process around subjectification enables a perspective on “the ways in which a human being turns his or herself into a subject” (Foucault, 1982, p. 327) Governmentality evolved according to processes by which government achieved its aims through the “conduct of conducts”. Using a Foucauldian approach, Miller and Rose (2008) carried out studies in clinical therapeutic settings, which were “laboratories of governmentality.” They adopted a Foucauldian focus on subjectivity and considered how this is produced both in personal and in impersonal domains through schedules, work and accounting systems, which become forms of power that operate beyond the state (Miller and Rose, 2008, p. 10). In their analysis, neoliberalism “saw the birth of a new ethic of active, choosing responsible, autonomous individual obliged to be free and to live life as if it were an outcome of choice” (Miller and Rose, 2008, p. 18).

Bourdieusian symbolic violence is power wielded through the symbolic: signs, symbols, language, discourse and pedagogy, the assigning of inferiority, and the denial of resources (Webb et al., 2002). Symbolic processes include how people are labelled and othered through classification and codification. In social spaces there are constant reciprocal acts of unconscious classification of practices, through which status groups, such as social classes, are formed and coded, creating clear symbolic boundaries, legitimising some people and practices and delegitimising others (Bourdieu, 1984; Webb et al., 2002). These contribute to a symbolic order that perpetuates symbolic violence. A key feature of symbolic violence is misrecognition, whereby a person misrecognises the situation as the norm. As this article will show, symbolic violence emerged powerfully throughout the policy processes under study.

In drawing together these theories to understand the parents’ experiences, the most contentious might be the integration of Marx, whose theory is often characterised as positivist, reductionist, structuralist, overly focussed on economic and labour relations, and lacking intersubjectivity and reflexity. However, Marx’s thinking considered the importance of social meanings, language and ideas; for example, he stated in the *German Ideology* that language and consciousness only exist in relation to other human beings: “it is man’s consciousness of the necessity of association with individuals around him, the beginning of consciousness that he is living in a society at all” (1885/1998, p. 50). Marx illuminated the psychosocial processes of alienated labour. A contrast might be drawn between structural thinking and the social constructionist approach of Bourdieu. However, Bourdieu, provides a bridge between structural and social reproduction in everyday life through a focus on practices and the power of a symbolic order and violence (Webb et al., 2002).

### Objectives

(1) To understand the lived experience of parents (as moral associates of children’s stigma) as they interact with food policy.
(2) To explore how parents resist stigmatisation.
(3) Reflect on implications for policy and practice.

## Materials and Methods

This study uses critical research and a qualitative approach to explore and understand the lived experience of parents as they interact with policy processes and their actors: policymakers and implementers. It is set in the context of the local state as a nexus of power relations that manage food policy, public health and local democracy. This provides a bounded terrain for research to explore the parents’ social world, food neighbourhoods or foodscapes, and other key influences in the context of power. It was carried out over 18 months during 2013 and 2014, in a London borough with a high prevalence of deprivation and “child obesity,” and it was organised into two phases: the first phase focussed on policymakers and implementers, and this informed the second phase by providing a local context, including the role of the local state, for the experience of parents. The core concern was to understand the parents’ experiences in context. Other policy actors served to triangulate these findings. The data was thematically analysed. Credibility was further addressed by a study report sent to parents, and feedback was invited.

Semi-structured interviews were the main method of data collection. The interview aimed to be an active process that encouraged the participants to explore perspectives, to conceptualise and to make connections (Holstein and Gubrium, 1997). The researcher is part of this active knowledge construction, so it is important for the researcher to reflexively consider positionality, power and bias. This involves having an understanding of participants’ social realities and of the researcher’s own insider/outsider positionality. The researcher in this study is working-class and has experience of poverty and community activism; however, it was not taken for granted that these would suffice to make the researcher an insider. Positionality became blurred. The researcher’s position as a dietitian and academic – positions that contain power – conveyed an outsider status to parents, but an insider status to policymakers and implementers. To reduce the potential for resulting biases, attention was given to reflexive field notes that considered power dynamics during interviews, and changes were made to subsequent interviews. A further attempt was made to observe society from the participants’ points of view by integrating ethnography into the study methods; thus, the interviews were ethnographically informed. This also contributed to the triangulation of data. Immersive techniques involved community observations that used audio recordings, extensive field notes and photography. Throughout the study, the researcher travelled by foot or public transport across the borough, noting observations of people and foodscapes, of food deserts and urban developments, which were sites of regeneration and gentrification. Photographs of foodscapes (not people) provided visual detail of what might be overlooked: the density of fast-food outlets, and the contrast between shopping parades in deprived and affluent areas.

The two topic guides (see Supplementary Appendix 1) were informed by the literature and colleagues in the field, and they were piloted. Key questions included icebreakers that asked parents: “One thing about childhood obesity that is important to you, anything at all?” and “Shall we use term overweight, obese or other?” Other questions included: “Thinking about what government says and does, are they helping or hindering parents?”, “Thinking about how food decisions are made, …Are parents involved – how could they be involved?”, and “What would you do if prime minister?”

Stimulus materials were “word cards” developed from key words and phrases used in food and obesity policies. Participants were invited to use these and did so in various ways. For example, some would choose one or two topics to talk about, whereas others used triads to draw contrasts. Public health posters and photographs of local foodscapes (ethnographic data) were also used (see Supplementary Appendix 2).

### Participants

The sample was purposive, and the recruitment strategy used convenience methods and snowball referrals. Recruitment was desk-based for the first phase. The sample frame was drawn up based on inclusion criteria of policymakers and implementers being involved in child obesity or food policy and delivery (community nutrition workers, obesity service and food providers). Potential participants were identified from local government websites and documents, including minutes of relevant committee meetings from the previous 18-month period, and they were invited to participate through electronic and postal communication. Sixteen participated: six policymakers and ten implementers (Table 1).

**TABLE 1:** Summary of policy actors: research participants.

**Policy actor groups**	**Definition/inclusion criteria**	**Sample**	**No.**
Policymakers (local government)	Position in local government with interest or direct involvement in child weight management	Elected representatives, including high level	6
Policy implementers	Role in delivery of food-related obesity policy	Range of community nutrition workers, senior management, chief executives and local business	10
Policy recipients	Parents/caregivers of children with obesity, aged 2–15 years	Mostly working-class (13):12 mothers and 1 father, middle- class (2):1 mother and 1 father, across range of ethnicities.	15

The recruitment for the second phase (caregiver/parents) involved face-to-face intercept at key community sites that had been identified during the activities of the preceding phase. These sites included community centres, housing offices and major workplaces (bus garages, supermarkets, local government). Requests were made to managers to advertise the research material and the researcher offered healthy-eating advice to staff. For example, a bus garage advertised the research on its electronic noticeboards, while researcher set up a health promotion table in the canteen; this yielded two recruits. Similarly, a table was set up in a housing office. With permission, the researcher was based in and recruited from community venues, in working-class and middle-class areas, such as in cafes and children centres. Health sector referrals were excluded to avoid a treatment-seeking sample that could introduce bias.

The sample frame considered a range of responses from different communities, ethnicities and social classes. Parents had children aged between 2 and 15 years who had been classified by a health professional as “obese”; this data was given by the parent, and the researcher verified the classification. The researcher did not directly measure children because this might shift the focus of the study from parents’ experiences of food policy to the children’s BMI. Participants lived or worked in the borough and were defined as working-class or middle-class according to occupation (Clement and Myles, 1997; cited in Scambler and Higgs, 1999) and the neighbourhood deprivation score. Parents were excluded if there were underlying medical conditions that promote child obesity. Following initial contact with the researcher, a screening tool confirmed the qualification to participate. Participant characteristics were collected by questionnaire. The information sheet and consent forms were given to participants prior to interview. Following their interviews, participants were asked to “snowball” referrals.

Interviews lasted for up to 1 h, apart from three that were longer. The interview process began with a confirmation of qualification. The interviews were either one-to-one or with a small group, depending on the preference of parents. Of the 15 interviews, 11 were with individual parents. Interviews were carried out at a place of convenience for participants and childcare was provided. As a thank you, dietetic advice was offered to families after the conclusion of interviews.

Of the 15 parents, 13 were mothers aged between 23 and 54 years. Seven were lone parents and 13 were working-class. The range of occupations included bus drivers and full-time caregivers in receipt of welfare (Table 2). Ethnicity ranged across nine groups, including Black African and Caribbean.

**TABLE 2:** Parent-participants’ characteristics.

**Parent characteristics *n* = 15**	
Age range (years)	23–54
Gender	13 females, 2 males
Ethnic group	1 Russian/Azerbaijani, 3 Black/African, 2 Turkish/Cypriot, 2 White/English 1 Black/British, 1 White/Black Caribbean, 1 Pakistani/Arab, 2 Black/Caribbean, 1 Asian/Caribbean, 1 other
MSOA – Index of multiple deprivation	13 reside in deciles 1/2 (high deprivation), 2 in deciles 5/6 (low deprivation)
Occupation	4 childcare workers, 1 adult-care worker, 2 bus drivers, 3 administration, 1 nurse, 1 teacher, 2 full-time homemakers, 1 unemployed
Education	13 secondary level, 2-degree level
Household	7 one-family lone parent, 7 one-family couple, 1 not say
State support	7
Housing tenure	11 social, 3 home owners, 1 not say
Social class	13 working-class, 2 middle-class

*Child data reported by parents: Age range: 2–15 years. Child BMI all above 98th centile.*

### The Data Analysis and Interpretation

The data analysis was thematic (Braun and Clarke, 2006), and the theming process was both inductive and *a priori*. The latter acknowledges researchers’ “prior theoretical understanding of the phenomena under study” (Ryan and Bernard, 2003, p. 88). The analysis plan began with analytical memos which were written following interviews, when transcribing and during subsequent readings. Transcripts were analysed in hard copy, with results transferred to NVivo QSR International Pty Ltd. (2014) for data management. The memos informed codes, themes and interpretation (Saldana, 2009). The first reading recorded initial thoughts and the second employed scrutiny-based techniques that looked for metaphors, transitions, repetitions and indigenous typology (Ryan and Bernard, 2003). Codes were formulated from a mix of the participants’ own words and the researcher’s conceptual understanding. A systematic approach involved a first stage of coding that used an “eclectic” approach of four coding methods: process, versus, descriptive, and *in vivo* (Saldana, 2009). In stage two, “focussed coding” methods were used: this involved combining initial codes to form concepts, look for connections and establish the major categories and themes. Mind maps were used to explore connections. As themes emerged, it was possible to think about theory for later interpretation. For example, the relevance of the Foucauldian approach to power and Bourdieusian symbolic violence of foodscapes became apparent during the coding process.

Field notes and photographs, as researcher-generated data, were coded and themed in the same ways as the interview transcripts, so they served to triangulate the findings and give them greater credibility.

### Results

**FIGURE 2:**
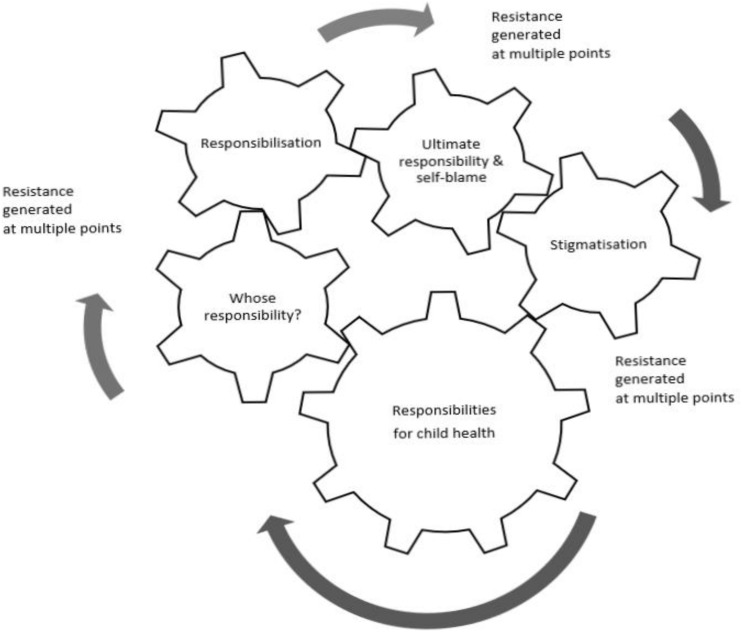
Theme of ‘Responsibilities’: a potential process of a large cog turning the small cogs of sub-themes.

In exploring the lived experience of parents as they interact with food policy, five core themes were inductively identified. The major theme of “responsibilities” is presented as a dynamic process in interaction with four sub-processes. It emerged as a powerful ideology around child welfare that backgrounded and interconnected with policy actors’ thinking and actions. The data shows contradictions and dissonance in how policy actors perceive their responsibilities. These are identified as ‘whose responsibility?’ and presented below as ‘views’ in subsections: policymakers; policy implementers and parents. The parents’ views progress to set out processes of responsibilisation, ultimate responsibility and self-blame, and of collective care and resistance. Figure 2 illustrates the interconnections: a possibility for how these discursive processes interact. The ideological views of individual responsibility lead to discourses of diffusion of responsibility, and processes of responsibilisation which can lead to blame shifting away from policy makers, governments, and industry and toward parents. Blame shifting can then lead to stigmatisation of parents, parent self-blaming and internalised stigmatisation among parents. Throughout these discourses there can be ambiguities and spaces for resistance. As posited by Sum, instability between discursive justifications and reality provides space for resistance through challenging, rejecting and transforming, creating alternative conceptions, and counter-hegemonic subjectivities (Sum, 2012, p. 2).

### Responsibilities

The universal concern for child welfare drives the motivation that was captured by the theme of “responsibilities.” It is a powerful idea; in this research, its presence was strongly felt and cut through the data. It emerged in processes that unfold through food policies across multiple sites and media, such as in policy literature, local state planning, language, and the foodscapes that impact on parents as the recipients of policy. The welfare of children was of paramount concern to all participants. This concern appeared embedded in discourses of healthy and unhealthy foods and body shapes, and thus in stigmatising processes that distinguish between “self” and “others,” and that cast parents as moral associates. For example, it was articulated in blaming parents, with assumptions that parents of higher-weight children lack caregiving competencies and feed children “unhealthily.” Data from the researcher’s field diary reflected these commonly held assumptions and provided insight into the embeddedness of stigma in communities:

Two mums followed me to give their opinion of parents of overweight children, saying “parents are responsible for feeding children properly” – “healthily” – “I don’t receive free school meals or benefits and manage” – “people expect to be spoon fed all their life”. And they argued it is possible for parents to cook healthily and inexpensively.

These high emotions around child health were common and further illustrated by a policy implementer who used the term “killing your child.” This is a powerful metaphor that constructs parents as a risk to child health and as the problem, while positioning the health worker as the expert who saves the child. This is read as a well-meaning anxiety, but it also exemplifies a divisive concept aligned with food and health illiteracy. Anna, a health worker, commented:

*Wife was definitely*… *overweight. The elder boys were thin. The young guy*… *seriously overweight*… *The daughter was too, and they took pride in feeding her. There was no way I could have a conversation. It was a badge of pride, they showed their love by giving her more things to eat. There was no way that they wanted to hear*… *the idea that you might be killing your child.*

In contrast, parents’ concern for child health was “taken for granted,” and surprise was commonly expressed that the researcher even asked about this. Most parents volunteered for school or community projects despite the pressures of employment and family. Thus, insight was gained into a collective obligation to care alongside that of the individual. Liz, a bus driver, explained:

*In classes at the* [community] *centre, we try to teach them about healthy eating*… *but it’s us doing it who are volunteers. When parents used to be able to do things with their kids because they had the time to do it. Whereas nowadays they haven’t, and I think that’s the biggest problem*… *too busy working.*

This bears significance because time-poor working parents volunteer to maintain services that would otherwise close due to spending cuts. This example concurs with the universal concern and contradicts the ambiguous thinking of other policy actors, according to which working-class parents lack competencies and are irresponsible. However, within this overall concern and responsibility for child health, there were tensions in the meanings and attribution of responsibility and what this means in practice: “who is responsible – the policymakers, food industry, implementers and parents?”

#### Whose Responsibility?

##### Policymakers’ views on responsibility

Among policymakers, there were contradictory views. On the one hand, “everyone is concerned” and wants to protect children; on the other hand, central government demanded that local policymakers make spending cuts, which led to compromises that do not protect child health. Policymakers described their responsibilities to central government and parents, and they adopted a policy of mitigation in attempting to comply with legislation while limiting the severity of funding cuts. For some, this was a cognitive dissonance as compromises were made between the interests of government and those of parents, and they appeared to distance themselves from the consequences, whether intended or not, of their actions. For example, Angie, a policymaker stated: *“We’re*… *constantly getting cuts and cuts. It’s about trying to mitigate the cuts rather than*… *do as much new stuff as possible.”* The crisis local government faced was elaborated by Ken, who said:

*We haven’t implemented all of the savings and the cuts that we’re going to need to*… *about eighty-two million pounds worth of savings so far. We’ve another eighty-five million pounds worth of savings to make that takes us to 2016/18.*

In contrast, Joe, a policymaker, countered the contradictory stance of colleagues in passing on cuts, framing it as hypocrisy:

*People of* [Labour] *political background would have voted for raft after raft of cuts to people who are the most socially disadvantaged.*… *So for me it all feels a bit sort of hypocritical that they can talk about food poverty but they’re not doing anything to really ameliorate that!*

There was ambiguity among local policymakers about their responsibility for the composition of foodscapes in which fast food outlets proliferated in deprived areas, unlike in affluent areas. Policymakers argued that the local state was *de facto* powerless, which presupposes no responsibility. They described urban planning as a permissive system that grants requests if they meet planning criteria. This lack of perceived power distanced policymakers from their decisions that had overseen the proliferation of fast food outlets. For example, one policymaker articulated the view that it was a “*chicken and egg*” situation, suggesting that low-income communities might want fast food outlets. This presupposed that deprived communities have power, choice and control over foodscapes; it also indicated “victim” blaming of communities and parents. Consequently, responsibility for providing nutritious foods in poor communities would not lie with the local state; instead, it lay with market forces and parental choice. Although some policymakers distanced themselves from their power in urban planning, they expected parents to exercise personal responsibility for food purchases. There was empathy for those in poverty who ate foods described as “*revolting*” and that would only be consumed if there was no choice. This is illustrated in comments by two policymakers, Mary and Ken:

(Mary)

*The other one I can’t bear besides McDonald’s is Iceland*… *It is the deprived who are going to Iceland*… *They have frozen cheese on toast. You just shove into the microwave*… *and they’ve got additives*… *It takes 5 min to make cheese on toast. It’s shocking really that people pay money for that*… *The very deprived are trapped into that sort of food.*

(Ken)

*There* [is] *connection between low pay, poverty and poor diet*…*cheap food is processed food*… *unfortunately, those foods, because of the industrialisation of food, are all too available*… *Some of us wouldn’t look at those foods but maybe we would if we had less money and had less skill*…

Although the lack of food retailers that support health was acknowledged, blame was shifted to parents by the perception that they are food illiterate. The use of the deficit model of parenting was, for some, highly gendered. For example, Mary talked about the food literacy campaigning of a celebrity chef that neglected to focus on mothers:

I mean Jamie Oliver of course tried, starting with school dinners. He was very committed. He did not move on then to educating the mums which is what I think is needed.

##### Policy implementers’ views on responsibility

The sample included public health nutrition professionals from a range of provider organisations and roles: from management to the “*coal face*”. They had responsibility for delivering new ways of working that accompanied spending cuts and privatisation. Confliction and resistance were apparent, and views were often not clearly demarcated but fluid. They described their responsibility as technical experts to support policymakers and parents by providing evidence, using performance-related management techniques, and delivering interventions. As with the policymakers, there was a dissonance between the reality of spending cuts and the service needs. However, the critique of policy processes by some implementers, showed resistance and challenged the structural factors and ideology of blame. For example, Claire, an implementer, commented on the role of politics in health:

*this is political, you know there’s a mayor, an elected mayor, what I became*… *aware of, is that its politics before health. So*…*there’s only certain things you follow, decisions are made on another basis*… *I’m not saying they’re necessarily political but I think politics is linked to how they’re voted in*… [it’s] *what they see rather than maybe the evidence base*… *and it’s very much who you know as well*… *it’s a real shame*

Some argued against blaming parents, instead framing parents in poverty as intelligent and resourceful. Bev, a community implementer, said:

*Blaming parents, for giving children food they are going to eat!*…*The most important thing we have is our energy. That’s the one we die without. To prioritise your energy at the lowest possible price seems to me, to be a really intelligent response to feeding children*… *Parents tell me*… *I can’t afford to waste food. I have to give children the food I know they’re going to eat. If you change the food of your family, and you risk waste*…

##### Parents’ views on responsibility

Parents’ views and experiences are read as having little power in a process dominated by national government and the food industry. The food environments – the supermarkets and the local retailers – provide them with few food options. Choice is determined by affordability. By virtue of food being sold, it is assumed to be healthy. Parents were unanimous in their views that government was neither helping nor meeting its responsibilities. Most thought government blamed parents and had a mutually supportive relationship with the food industry, as exemplified by Andrea, a mother:

*With one breath, the government are blaming those outlets*… *with the next breath – because they make the money from the shops – they’re allowing it to happen.*

Parents talked about their cooking skills, that food compromises were made when tired or stressed, and their distrust in manufactured foods, and they questioned the motivations of the food industry and government. Felecia, a mother, commented:

*They’re* [the government] *not helping, I love cooking and find it better to cook at home*… *when tired I go to fast food shops, can’t be bothered to cook. But I like to cook stuff at home so I know what’s going in. I see my kids growing up*… *fast foods popping up everywhere. I feel the government is allowing all these shops to pop up a couple of yards away from each other, just to give you quick food.*

Many parents described their situation as subject to powerful forces that constructed their food environments and over which they had no control. This may be read as either disavowing responsibility or as lived reality in the face of political and structural constraints. Either way, most parents were aware that they interacted with other social forces. Bedria, a mother, commented:

*Its*… *the economy*… *and government, everything linked together*… *It’s one big chain goes around and we’re in the middle.*

Parents spoke about the responsibility of local government in relation to fast food outlets. Their proliferation was assumed to be because they provided an income stream for local government. Parents challenged how and why so many outlets were given permission to open in deprived areas and around schools. Khadra, a mother, said:

On every corner, there is a chicken easy shop. They are cheap. I don’t think that’s very helpful. While children coming from school, they buy French fries or chicken. Not helpful to give a license to everyone.

Parents believed that the financial interests of the food industry and government took priority over child health. The word “*allowed*” was frequently used to describe the relationship between government and food industry, and parents articulated that certain food products “*shouldn’t be on the shelf*” and that food was “*all about money*” and that “*they make fast food easier*”. There was anger that this leads to the production and sale of foods that are unhealthy for children. Parents thought that the food industry was not taking responsibility. Food advertising was described as ubiquitous; it was “*like a radio – it’s on all around you*”. There was distrust and cynicism that the government was choosing not to act, and parallels were drawn with tobacco control. Cynicism was exemplified by Leyla, a mother and childcare worker:

*government*… *if they put a shut down on what happens, on smoking or whatever, you will see a cut down drastically*… *if they wanted to make a change they could, but they’re choosing not to.*

Parents’ policy solutions included clear food labelling and product reformulation, a stop to the manufacture of unhealthy foods, and the accountability of the food industry. For example, in talking about the Responsibility Deals (Department of Health, 2011) – the legislation based on voluntarism of the food industry – Andrea, a mother, commented:

It shouldn’t be voluntary. There should be certain stipulations that these products come up to. It should be illegal for them to not be doing what they should be doing. Like it’s illegal for me steal from somebody. Why is not illegal for them? They’re being allowed to get away with it. It should be a criminal offence. People are eating this muck!

At the same time as challenging the ethics of the food industry, some parents voiced a fatalism about the food industry’s domination. Syrita, a mother, said:

*They’re a business. So, as I said, supply and demand*… *They can see that if a child wants this*… *then they’re going to go for it and either make it that bit cheaper or that bit sweeter*… *to entice the kids.*

In this fatalism, there is an awareness of exploitation and discrimination. This mother’s testimony later described her maternal sacrifice that involved reducing her own food intake to provide fresh chicken and salad for her child.

#### Responsibilisation: ‘We’re Getting the Message’

Most of these parents were aware that government actions increased their parenting responsibilities, and they expressed cynicism toward government. This was articulated by Andrea:

*We are getting the message, but they still don’t seem to be doing anything about it*… *still allowing all these products to be sold because you want the revenue from them.*

“Getting the message” relates to the process of transferral of responsibilities by means of convincing parents of the need to change their childrearing behaviour to help child weight management. The evidence that parents were receiving the message was illustrated in their language that embraces behavioural change – “*discipline,” “monitor,” “regulate,” “reading labels*” – and the moral imperative of knowing right and wrong foods. New responsibilities were being created that were in tension with the social reality of material constraints and cultural and class differences. The following quotes suggest that the language of skills-based parenting is part of the everyday language of working-class parents. However, it is socially divisive among parents with higher-weight children, as well as among many of those whose children are categorised as “normal weight”. Lena used social learning terminology to contrast the everyday practices of her working-class community:

in area of lower class, people just do what they do without thinking, shaping and monitoring. They just live.

Kerry, a father, suggested the need to chastise other parents:

*when you see a child who is very overweight, you look at the parents and say “Why haven’t you tried to regulate him and reduce his weight?”*… *tell him he can’t have this and can’t have that*… *it’s very important.*

Judgement was expressed by some about parents’ food choices. Leyla stated:

*you can choose what you buy from the supermarkets*… *as adults should know what’s right and wrong.*

This parenting discourse framed what is normative, although it was contradicted by the classed realities of necessity and “no choice”. Leyla commented:

*They can afford to go out and buy these organics, healthy foods*… *have nannies that prepare the dinners before they get in*… *told the nanny “make sure you feed them healthily”. But when you’re thinking every day, what am I going to cook them? Your money’s running low. You’ve got stresses about bills and everything else. The last thing on your mind is “what’s the healthy option?” You can’t afford to buy the healthy stuff so you’re just going to go for the quick fix.*

The social division in “knowing” of difference in resources is represented by the “nanny.” The knowledge of difference was apparent in everyday lives as affective injury relayed by foodscapes in deprived areas, as illustrated by Leyla, who described the composition of her high street:

*it’s keeping the adults on their liquor, the kids on the sweets and then the take-aways for dinner*… *It’s what we’re seeing everyday so all we think about is sweets and drinks*… *It’s like the betting shops. a lot more people are doing it*… *it’s not good.*

The message relayed through the foodscapes was seen as devaluing their children’s health. As she looked at a photograph of a supermarket in an affluent area, Felecia, a mother, commented:

*Now that looks pretty. It looks like that would be more healthy. it looks like a little health food shop*… *it’s not life threatening.*

A further mechanism in relaying a message to parents was the National Child Measurement Programme (NCMP), a programme that measured schoolchildren’s BMI and informed parents of the result by letter. The NCMP entered the arena of socially embedded stigma that cut into families and communities and was layered with social class and poverty. The stigma attached to parents as moral associates was being backgrounded by safeguarding legislation and policies, such as the NCMP. Leyla described the impact of the “letter”:

*when you get the letter of your child’s measurements you assume it’s the parents’ fault*… *parents are going to talk. People are going to talk and assume that the parents are obese as well. Or you know, neglecting the child. Don’t care. Just feed it to shut it up.*

The symbolic power of this message is validated by the earlier reported notion of “killing the child,” which was expressed by a policy implementer and tied to the notion of safeguarding. “Killing the child” is read as a message about the knowledge of health risk and preventative action on the part of parents. This exemplifies fear-based messaging that uses the threat of chronic diseases to nudge behavioural change. As the message is received by parents, subjectification occurs as they self-constitute as neoliberal parents who carry out policy requirements. Samina, a young mother in receipt of welfare, used the epidemiological language of risk:

*They do say it’s a disease*… *scary. I want my children to be healthier. I know it’s dangerous for their health. It’s a health risk.*

This subjectification of becoming the neoliberal parent is played out through the performance of practices, which is a process involving self-judgement against the social norm. Paradoxically, as parents become aware of the health risks, there is a feeling of discrimination. In “getting the message,” they know their children’s lives are devalued. Yvonne commented:

*We don’t cost anything when they bury us*… *They never suffer.*

The feeling of being devalued was relayed through comments on the material reality of the food environment. For example, Maya said:

They dump those things in our area because they see it as deprived and they think the people who live there don’t matter.

#### Ultimate Responsibility and Its Social Implications

The data reveal an overwhelming presence of responsibilities to protect child health, with tensions in attributing responsibility and the constant emergence of stigma. The internalisation of stigma was articulated as “*ultimate responsibility.*” This phrase was used by all the parents who assigned self-blame. The social implications of this internalisation became clear as data showed collective blame for parents, which led to the blaming of others and social division. Stigma was consented to through performance, and it was resisted by challenging the policy discourses and by actions of unity, by collective care for children, and ultimately by the policy solutions.

Performativity and guilt were powerfully illustrated through parents’ self-reported practices. In self-blaming, many parents used the language of performance, such as “*can’t blame someone else for what I do,” “food on the plate,” “in the cupboard.”* Most were aware of the powerful influences around the food system, yet they took ultimate responsibility by performing the food duties of taking from the shop shelf and feeding the child. Bedria, a childcare worker, said:

*we’re the one who just picks it up!*… *It’s us who’s responsible for what goes into my child’s mouth.*

In using the language of self-regulation, parents constructed the parent-selfhood of what they should be. They were engaged in a cognitive struggle as they compared themselves with others and internalised blame. This is illustrated by Ferda, a mother, who participated in a community weight management programme for children based on behaviouralism. Although a remarkable cook with a healthy Mediterranean tradition, she criticised herself for not having sufficient control over her child’s eating in comparison with her neighbour. Along with her self-blame for her perceived lack of control over her child’s diet, she indicated that there are challenges in children accepting prescriptive approaches to diet. She stated:

*When the parent goes to buy the food they should not get what they* [the children] *want but do the healthy food, or see if they will eat it or not. But in my case if my ones don’t, that’s very difficult. But I think other children would if, you know, they were on like a schedule. Because our neighbour*… [child] *not allowed chocolate and things like that. They’ve got to have a certain cereal in the morning. They can’t have no snacks during the day. It’s all healthy food. Vegetables, fruit and then they have the main dinner*… *but it’s well controlled*…*very good control and they eat all very healthy*… *she’s done a well job for them.*

Ferda established difference by stereotyping her neighbour’s good maternal control, compared to which she self-stigmatised as a “bad mother”. Ferda also described her lack of financial resources and her maternal sacrifice to feed her children:

… *parents have control*… *can’t control the whole 24 h*… *don’t give them pocket money to get that kind of stuff and give a proper meal at home. But*… *you might not have no food in. You got to compare everything with your situation*… *how people are living. have money but then maybe they run out. They paid the bills, and they haven’t got enough for shopping. I’ve been in that situation and I know it’s very difficult. I pay all my bills first*… *whatever’s left will go to shopping. Some days I don’t have nothing, and I find it difficult. If it was just myself, that would be fine but when you got kids, they want all the time, so you go to you know*…. *with me, is always kids first. I will go without.*

Social divisions emerged because, in the context of taking ultimate responsibility, the attribution of self-blame was collective. Parents blamed themselves and other parents, and they were blamed by parents of “normal” weight children. Stigma was also attached to welfare recipients who wanted to spend time with their children – that is, they were caregiving – which points to the imbalance between family and working life. Working parents were forced to make food compromises as part of the negative externalities of work. Parents articulated these externalities as resulting from lack of time. Liz, bus driver and mother, argued that working parents had less time for caregiving, with the result that cooking was elevated to quality time:

There are people on benefits in this area who’ve got a good quality of life with their kids because they are at home and are able to cook. I think it’s more the working parents that are suffering and the kids of working parents who are suffering.

In contrast, Felecia, a mother in receipt of welfare, resisted the stigma and argued that she had the right to raise her own children. She articulated a counter-argument to the political economy of neoliberalism in which the state supports a commodification of childcare to increase the workforce, which is part of the neoliberal notion that citizenship is based on paid work (Williams, 2005, p. 28). Felecia considered it economically illogical that mothers are forced into work so they can pay someone else to raise their children:

*when you’re on benefits, they feel you squander it. You’ve got a roof over your head, paying your bills, doing your shopping, feeding your family as best you can. It’s not life-changing money you’re getting, its money just to live*… *stereotype people who are on benefits, not worthy*… *very unfair, because sometime is not your fault, certain circumstance. You want women to have children and go back to work. Who’s going to raise their children? Then why should you have them? Why should you pay other people to raise your children? That doesn’t make sense. I decided that I was going to raise my children. Yes, I was on benefits.*… *I don’t want my children to go childcare and the government helps me pay for it. Why? I don’t need them to do that. I will do my bit and look after my children because I had them, you see.*

Paradoxically, parents were blamed for lack of care, yet they desired to care more. This appears to be a resistance underpinned by a rights discourse: the right to raise children. Resistance was articulated as anger at the government and the food industry, whom parents perceived as colluding in the interests of the market economy.

### Collective Ethic of Care and Resistance

Although, in taking ultimate responsibility, parents self-blamed, they also faced common challenges and shared experiences that united them. A key concern for parents was the stigmatising effect of the word ‘obese’ and the deleterious effect this stigma has on the child’s well-being. All volunteered in communities, mostly as a result of cuts in council spending. This suggests a collective ethic of care that was reinforced through the policy solutions of parents. These tackled the work–life balance and, at the community level, argued for control of high-street planning, in order that high streets support health and family life. Parents advocated greater control of the food industry so that healthy foods would be the norm in all communities, and they suggested that the food system be fundamentally changed. The parents’ policy solutions diverged from those of the United Kingdom government, notably in their argument that there should be community involvement in food policymaking. Some went further and argued for political involvement. A summary of their policy suggestions is as follows:

(1) Employment and welfare reforms, including improved working conditions to support childrearing, food vouchers in or out of work.
(2) Greater control of food industry including mandatory “responsibility deals,” advertising restrictions, product reformulation, affordable nutritious foods, avoiding increasing food costs through taxation, honest labelling.
(3) Focus on community and schools, including family eating clubs, redesign of high streets with small retailers and removal of most fast food outlets. In schools: no targeting, nutrition on curriculum, universal free meals, and cooking lessons. Schools and community venues as spaces for parent–peer support, and policy involvement.

In essence, policy solutions diverged from the *status quo*. Parents were not passive policy recipients; rather, they articulated food democracy and sovereignty. Change was articulated by two mothers as a “*food revolution*.”

In summary, responsibility emerged not as a singular, linear process, but as multiple, interconnected processes that cut through social lives. Amid concern for child health, responsibility was found to be diffused and ambiguous. The government and food industry were regarded as being irresponsible. However, in a context of stigmatisation, parents self-blamed; at the same time, they participated in collective care in their community as services were cut. Resistance was shown through their anger and awareness of discrimination, and ultimately in their policy solutions.

## Discussion

The findings show how the notion of responsibility is central to parents’ lived experiences as they interact with food policy. It intersects their lives on multiple levels with tensions, ambiguities and contradictions. Using critical theory provides an understanding of how the findings relate to processes of power, stigmatisation as a social process, and how caring responsibilities are distributed according to neoliberal rationalities rather than by meeting social needs. There are important implications for policy and practice. The findings are consistent with existing literature and theories.

The importance of child welfare was omnipresent among policy actors. The social power of this idea is theoretically treated using the Marxist “ruling idea” of universal “common sense”, which exists independently but in actuality conceals the relation of domination (Marx and Engels, 1845/1998). This is a concept used by Mahoney in relation to the notion of individual responsibility for consumption and diet-related health (Mahoney, 2015, p. 47). Gillies et al. (2017) argue that the contemporary “child saving” movement in the United Kingdom is the taken-for-granted thing to do, but that it veils the contradictions in the pro-market system according to which children are exposed to harm rather than protected. This perspective does not underplay the right to good health and the flourishing of children, but it points to the contradictions. Instead, it has a historical context exemplified by the 18th- and 19th-century child rescue movement that rooted child maltreatment in poverty and parent irresponsibility and which, according to Evans et al. (2008), was a means to regulate deviant populations. Furthermore, in present society, health has become a regulatory discourse of “child saving” that uses the language of crisis to shape social norms.

The findings relating to “whose responsibility?” concur with both Tronto (2013) and Gillies et al. (2017), in that the diffusion of responsibility confuses where responsibility for care resides. Although, according to policy, everyone is responsible, the lived experience of parents was that government colluded with the food industry to produce and distribute foods harmful to child health. Ambiguities reflected the diffusion of responsibilities and provided space for attribution of responsibility to others, and thus for the acts of blaming and stigmatisation. The political context for the ambiguities around responsibility echo Tronto’s contention that

politics [is] about making judgement of the relations that exist and how needs might be met… that politics involves meeting needs in a way that permits the pursuit of other goals as well, and … it involves making decisions about who does what for whom. (2014, p. 49)

Confliction arose for policymakers who were charged with tackling child obesity, yet who believed that they had little power to resist spending cuts or to control the foodscapes that promoted unhealthy foods. In Tronto’s terms, policymakers were releasing themselves from responsibility through compliance; thus, they embodied a privileged irresponsibility (Tronto, 2013, p. 60). In passing responsibility to others, the policymakers reduced their own responsibility. This was not a passive process; rather, it involved hegemony and governmentality – that is, the soft power that wields stigma.

In a process of Gramscian hegemony, the local state was seen to act as a transmission belt for central government, and this was contested: not all policymakers and implementers consented or complied, since some questioned, challenged and resisted. Subjectivities were being constituted and challenged through their reflexivity. The subjective positions of policy actors in the obesity terrain have been explored by Monaghan et al. (2010). These social theorists used Foucauldian governmentality to identify the construction of six subjectivities of actors involved in constructing the notion of the “obesity epidemic.” As found in the present study, responsibilities were performed by implementers in the enforcement of practices, such as data collection. The ambiguities of policy actors add to Monaghan et al.’s (2010) discussion, as some, for example, countered the stigmatising discourses and challenged the construction of food-illiterate parents. Caraher (2016), for example, argued that parents in poverty are highly resourceful. Parents constructed the entrepreneurial, neoliberal parent-self, with some actively consenting to take personal responsibility – a neoliberal construct – involving self-regulation, monitoring, disciplining and comparing with others leading to self-doubt and blame. This was also challenged by parents, as many stated that it did not correlate with the reality of their time and money constraints and values. As Bowen et al. (2014) found in a large qualitative study with largely working-class mothers in the United States, mothers were poor and time pressed, had the skills to cook family meals but resisted policies that glamourised cooking, because these were disconnected with their reality. These findings suggest a process of negotiation, of consent and provides insight into how counter-hegemony, provides space for ambiguities.

A further example of Foucauldian governmentality was illustrated in the message mediated through the NCMP. In Foucauldian terms, the measurement individuates and “others” the child and parent as moral associates. The letter was found to enter a stigmatised environment, and, against the background fear of child safeguarding, the parent was being marked out as neglectful. This study posits, therefore, that programmes such as the NCMP have unintended consequences that are counterproductive to engaging with parents. As in the present study, Nnyanzi et al. (2016) found that informing parents of the results by letter mediates stigma; parents prefer feedback through personal contact with health professionals. Others have found parents to be supportive of the NCMP, with only small amounts of negative feedback (Steventon et al., 2012) and a negligible stigmatising impact on children (Falconer et al., 2014). However, Falconer et al.’s (2014) study had low response rates, so sample bias may account for their finding. This article suggests that the NCMP may be abstracted from its social context of multi-layered embedded stigmas. In Bourdieusian terms, the process and letter become a symbolic violence that, albeit unintentionally, labels and devalues the caring practices of these parents. It is suggested that it leads to an affective injury on parents as moral associates, with social amplification into communities.

The parents interviewed in this research met their caring responsibilities and all took ‘ultimate responsibility,’ even though many clearly struggled with resource deficiencies. As Tronto argues, people cannot be blamed if they do not have resources (Tronto, 2013, p. 132). Furthermore, in allocating responsibility of care in society, there is a political responsibility as to whether or not those with the responsibility have the resources to function (2013, p. 55). The ambiguities among policymakers about their power in urban planning and the distribution of retail outlets that provide healthful or harmful foods illustrates the distortions of market forces in providing care (Tronto, 2013, p. 115) as well as their own roles in the management of the local state.

Insight was provided into discursive processes around fear-based public health messaging aimed at behavioural change. These are processes through which the parent embraces responsibilities to manage risk and prevent child ill health. Ramos Salas et al. (2017) point out that using the notion of ‘obesity’ as a risk factor promotes prevention policies rather than treatment. And, their critical policy analysis of obesity prevention policies, use of categories such as ‘healthy’ and ‘unhealthy’ weights contribute to stigma. From a Foucauldian perspective, fear-based messaging was the technology for behavioural change in the cultural sphere, taking the form of texts, images, ideas and the spoken word; the latter were the words of policy implementers and what parents heard every day. This concurs with O’Hara’s (2014) critical discourse analysis of Australian weight-related public health initiatives, which found a dominant discourse of “preventative health” was foundational for a number of discourses that are dissonant with the principles of health promotion. These included discourses of health motivation through “alarm and fear” (O’Hara, 2014, p. 222) and discourses of “responsibility.” Moreover, notions of risk have been argued to be ineffective, since risk conveys different meanings to different people: statistical probability; subjective and human risk (Speigelhalter and Blastland, 2013, pp. 4–5) and political risk as “a way of ordering social imaginaries” (Warin et al., 2015, p. 309). Risk confers short- and long-term meanings, consideration of which includes class-based parental resources and priorities (Warin et al., 2015). While fear and risk for future child health were articulated by some parents, they also described the more immediate concerns of everyday life. McKenzie (2012), in her study of working-class life on a Nottingham council estate, found that “women’s lives were full of risk management” in the everyday, and that they included stigmatisation (2012, p. 131). As with Garasky et al.’s (2012) research in the United States, the “everyday” in this data, included financial and environmental stresses that they found associated with obesity in children. These authors suggest that there is less control over food choices in such scenarios of poverty (2012, p. 127).

Symbolic violence leading to affective injury also related to the foodscapes in deprived areas. The shopping parades consisting of shops that do not support health conveyed a message of lack of worth to parents, in contrast to the health-promoting options available in affluent areas. The food outlets in deprived areas were not a community choice, as some policymakers implied. Mahoney (2015) has shown how the food industry targets post codes, social status and class in its marketing. The foodscapes in deprived areas produced feelings of poor physical and mental well-being, and processes of embodiment were described. This perspective on symbolic violence is of “the knowing”; that is, parents are conscious that they face discrimination through the food options available in their communities and over which they have no control. A similar sense of “knowing” but not having the capacity to resist due to life pressures was found by Atkinson (2017). The parents had not consented to this environment; on the contrary, they articulated that they had no control over or understanding of how fast food outlets had flourished. There was both fatalism in this feeling of no control and a counter-hegemonic space in which anger was voiced as resistance.

Self-blame was most graphically evidenced through the parents’ language of performance, which reflected their perception of themselves as having ultimate responsibility in their practices despite the constraints they were under. Thus, they combined self-sacrifice and self-blame. In Foucauldian terms, this is the process of becoming the neoliberal parent-self, which involves the subjectification of “social control not through physical force but the production of conforming subjects and docile bodies” (Parker and Aggleton, 2003, p. 17). In this process, the parent judges, normalises and others the self. It is a power process through which stigma and self-stigma are produced. This self-blame through performance is played out in the popular media in television programmes (Rich, 2011). By blaming themselves, parents were taking “ultimate responsibility.” Parents illustrated how they strove to fulfil neoliberal “personal responsibilities” through volunteering and competency-based care. Tensions arose as parents’ experiences evolved into bridging the contradictions stemming from inequalities in resources that often left them only with unhealthy choices, maternal sacrifice and stress.

Resistance to stigma and moral association were explored at the level of the parent-self by Davis et al. (2018), who found that stigmatisation is psychologically hindering as a result of self-blame, but that some parents utilise their own experience of body size to protect children’s sense of well-being and to limit self-blame. The present study’s findings indicate a social layer to parents’ resistance, whereby it was presented as both an individual and a shared experience of anger, as collective volunteering, and as articulated politically through policy alternatives that argued for material resources and greater control over foodscapes and the food industry. Paradoxically, resistance was politicised due to the fear generated by public health messaging in an environment over which parents had little control. There was a feeling, therefore, of discrimination and of their children’s lives being devalued. Feelings of discrimination and injustice were also found in Sealy’s (2010) research on deprived areas of the Bronx, where parents believed that more affluent areas sold foods of better nutritional quality. In addition, there were instances of classed resistance. These were voiced as collective feelings of discrimination and difference, but mostly not as “class.” Instead, community had a strong resonance with class, as did taking ownership of the local food supply chains. For many, dealing with the pressures of everyday life was paramount. As Atkinson argues, this constrains the possibilities of resistance or struggle (Atkinson, 2012, p. 29).

This research adopted a critical and transdisciplinary stance that supports an understanding of complexity, including in the political context. The study design and systematic reflexive approach to both study design and theory reduced the interference of bias. The multiple data sources, which enabled triangulation, worked well to support the study’s internal validity. The ethnographic preparation served data collection and aided “insider” positionality, which prevented the potential for bias due to the researcher’s past experience in community activism. Although an active interview stance was taken, to avoid bias the researcher’s voice was minimal and was reflected upon after each interview. A key question is whether the number of interviews was sufficient for the analysis. This involved considering whether the emerging themes were saturated and whether anything new was emerging from the data. The literature recommends a range of 1 to 60 interviews, with an average of 30, but the key is the generation of sufficient data (Baker and Edwards, 2014).

### Implications for Public Health

Using critical qualitative research with multiple theorists and methods has provided important insight into the lives of stigmatised parents as moral associates of children’s obesity, and has addressed how policy processes in different forms, whether of foodscapes or NCMP, interact with parents’ lives and mediate powerful messages that devalue and stigmatise. Stigmatisation through public health obesity discourses is documented with calls for reflexivity in policy and practice, and for a greater involvement of the lay voice to inform policy (Boswell, 2017; Ramos Salas et al., 2017, 2019). This study contributes to this literature through its insights into how individual or personal responsibility becomes ultimate responsibility in the form of self-blaming, diffusion of responsibility and responsibilisation. Whether or not an intended consequence of policy, this does not serve child health well or meet the policy ambition to reduce obesity prevalence. In the context of the social gradient, it could maintain the *status quo*. Given this, the following changes to policy and practice are recommended:

• Ending stigma by using health equity: stigma is mediated not just by people but through a range of policy sites, documents, and places, including foodscapes, so health equity should be integrated with local government, for example, in urban planning.
• Parents’ policy solutions: parents have indigenous knowledge of what impacts their children’s health and should be treated as “experts by experience.”
• Participatory health equity: processes that assess the health equity of policies should involve the expertise of parents in their lived environments.
• Social gradient: revisiting this index in order to include the meanings of the lived effects of class, stigma and discrimination. This would aid the reflexivity of practitioners and policymakers.
• Reflexivity of policy makers and implementers: to consider stigma as a social process involved in social divisions, and how practitioners might unconsciously be part of stigmatising processes.
• To consider obesity terminology, mindful of individual preferences and how the policy narrative could change to support health equity.
• Policy direction: public health policy needs to fundamentally shift from individualised behavioural change to tackling the structural factors of the social determinants of health.

Through this research, an understanding has evolved of the social realities of parents’ lives as they interact with food policies. The neoliberal notion of individual responsibility results in stigmatisation, and the internalisation of responsibility results in self-blame. Parents care for their children, but they are cynical about government and the food industry’s level of care. They experience a diffusion of responsibility, and they are responsibilised to make up for cuts to community services. Critical theory provided the tools for examining the power processes that influence parents to accept ultimate responsibility. Although accepted, this responsibility is also resisted. Against material constraints, parents blame each other, but under the surface is an argument for the social rights to care – that is, for the material resources to enable care. Despite social division, there is a collective responsibility among parents. This assumes a societal focus through the parents’ policy solutions and recommendations for advancing child health, which are based on their experiences. This may not be a fully formed community of resistance taking the form of political action in response to stigmatisation, but this research nevertheless provides insight into potential for such a community of resistance to develop.

## Conclusion

Critical qualitative research is underpinned by knowledge based on meanings, and it is context bound. In this case, the context is working-class parents living in an inner London borough. The participants reflected the area’s demographics of ethnic diversity, the prevalence of women as the main caregivers, and the poor working-class (both in and out of work) social composition of the borough. A shortcoming of this article is that it does not address the questions of intersectionality and gender. The research is not transferable, but this does not diminish its importance. Understanding the social realities of parents as moral associates of child obesity allows for the attribution of blame to be challenged; moreover, in the context of failing policies, it enables new ones to be found based on the experiences of parents who take “ultimate responsibility.” A deeper understanding of power processes involved in supporting political ideologies allows practitioners, policymakers and parents to consider alternatives that would reduce the social gradient in child health. Given policy failings, more can be learned about new policy directions by engaging with those who have expertise from experience – that is, the parents themselves. Future studies on changing the obesity narrative could explore forms of resistance, and how these might involve a new generation of food, body and health equity activists. Such activism could lead to policy changes that reduce stigma and promote equity.

## Ethics Statement

Ethics approval was obtained from the City University School of Arts and Social Sciences Research Ethics Committee. Interviews were confidential. Participants were assigned fictional names and they gave informed and written consent. Weight-neutral terms such as “higher-weight” were used, as was the terminology presented in policy or used by participants.

## Author Contributions

SN-G is the sole contributor who conceptualised and designed the study and wrote the manuscript.

## Conflict of Interest

The author declares that the research was conducted in the absence of any commercial or financial relationships that could be construed as a potential conflict of interest.

## Footnotes

1 Note on language: While child health is of paramount concern, it is recognised that the term “obesity,” its derivations, terms relating to categorisation of weight have contested meanings and uses. These are therefore placed in inverted commas when first used. The term “higher-weight” is used in recognition that not all bodies categorised as “obese” are biologically disordered.

## References

[B1] Atkinson W. (2012). “Economic crisis and classed everyday life,” in *Class Inequality in Austerity Britain*, eds Atkinson W. Roberts S. Savage M. (London: Palgrave Macmillan), 13–32. 10.1057/9781137016386_2

[B2] Atkinson W. (2017). *Class in the New Millennium: The Structure, Homologies and Experience of the British Social Space.* London: Taylor & Francis.

[B3] Baker S. Edwards R. (2014). *How Many Qualitative Interviews is Enough? Review Paper.* Southampton: National Centre for Research Methods.

[B4] Bissell P. Peacock M. Blackburn J. Smith C. (2016). The discordant pleasures of everyday eating: reflections on social gradient in obesity under neo-liberalism. *Soc. Sci. Med.* 159 14–21. 10.1016/j.socscimed.2016.04.026 27155225

[B5] Booth R. (2019). *UK’s ‘Cruel and Harmful Policies’ Lack Regard for Child Hunger, says NGO, theguardian.com.* Available at: www.theguardian. com/society/2019/may/19/uk-government-cruel-policies-child-hunger-breach -human-rights-says-ngo (accessed January 09, 2019).

[B6] Boswell J. (2017). *The Real War On Obesity. Contesting Knowledge and Meaning in a Public Health Crisis.* London: Palgrave Macmillan.

[B7] Bourdieu P. (1984). *Distinction.* Oxford: Routledge.

[B8] Bowen S. Elliott E. Brenton J. (2014). the Joy of Cooking? *Contexts* 13 20–25. 10.1177/1536504214545755

[B9] Braun V. Clarke V. (2006). Using thematic analysis in psychology. *Qual. Res. Psychol.* 3 77–101. 10.1191/1478088706qp063oa

[B10] Bresnahan M. Jie Z. (2016). Detrimental effects of community-based stigma. *Am. Behav. Sci.* 60 1283–1292. 10.1177/0002764216657378

[B11] Caraher M. (2016). “Food literacy beyond the individual: the nexus between personal skills and victim blaming,” in *Food Literacy: Key Concepts for Health and Education*, ed. Vidgen H. (London: Earthscan), 118–132.

[B12] Clement C. Myles J. (1997). *Relations of Ruling Class and Gender in Post-Industrial Societies.* Montreal: McGill-Queen’s Press.

[B13] Collinson A. (2019). *Real Pay Cut For Millions Of People In Working Class Jobs.* Available at: https://www.tuc.org.uk/blogs/real-pay-cut-millions-people-working-class-jobs

[B14] Davis J. Goar C. Manago B. Reidinger B. (2018). Distribution and disavowal: managing the parental stigma of children’s weight and weight loss. *Soc. Sci. Med.* 219 61–69. 10.1016/j.socscimed.2018.10.015 30391871

[B15] De Brun A. McCarthy M. McKenzie K. McGloin A. (2014). Weight stigma and narrative resistance evident in online discussions of obesity. *Appetite* 72 73–81. 10.1016/j.appet.2013.09.022 24096083

[B16] De Vogli R. Kouvonen A. Gilmeno D. (2014). The influence of market deregulation on fast food consumption and body mass index: a cross national time analysis. *Bull. World Health Oragn.* 92 99A–107A. 10.2471/BLT.13.120287 24623903 PMC3949530

[B17] Denzin N. Lincoln Y. (2011). *The Sage Handbook of Qualitative Research*, Fourth Edn London: Sage.

[B18] Department of Health (2011). *The Public Health Responsibility Deals.* London: Crown.

[B19] Department of Health (2018). *Childhood Obesity Plan.* London: Crown.

[B20] Dinsdale H. Ridler C. (2010). *National Child Measurement Programme: Guidance for Analysis by Public Health Observatories and Primary Care Trusts.* Oxford: National Obesity Observatory.

[B21] Ditlevsen D. Reventlow S. Nielsen A. (2016). From policy to reality: early overweight, structural barriers, and the allocation of responsibility in the Danish health care system. *Crit. Public Health* 26 566–577. 10.1080/09581596.2015.1153810

[B22] Dorling D. (2018). *Peak Inequality: Britain’s Ticking Time Bomb.* Bristol: Policy Press.

[B23] Evans J. Davies B. Rich E. (2008). The class and cultural functions of obesity discourse: our letter day child saving movement. *Int. Stud. Sociol. Educ.* 18 117–132. 10.1080/09620210802351367

[B24] Falconer C. Park M. H. Croker H. Skow A. Black J. Saxena S. (2014). The benefits and harms of providing parents with weight feedback as part of the national child measurement programme: a prospective cohort study. *BMC Public Health* 14:549. 10.1186/1471-2458-14-549 24888972 PMC4057922

[B25] Foucault M. (1975). *Discipline and Punish: The Birth of the Prison.* New York, NY: Second Vintage.

[B26] Foucault M. (1982). The subject and power. *Crit. Inquiry* 8 777–795.

[B27] Fox R. Smith G. (2011). Sinner ladies and the gospel of good taste: geographies of food class and care. *Health Place* 17 403–412. 10.1016/j.healthplace.2010.07.006 20801073

[B28] Garasky S. Gundersen C. Stewart S. Eisenmann J. Lohman B. (2012). Economic stressors and childhood obesity: differences by age and gender. *Public Health Soc. Behav. Health* 2012 115–132. 10.5772/37369

[B29] Gillies V. (2011). From function to competence: engaging with the new politics of family. *Sociol. Res. Online* 16 10.5153/sro.2393 (accessed June 05, 2018).

[B30] Gillies V. Edwards R. Horsley N. (2017). *Challenging the Politics of Early Intervention. Who’s ‘Saving’ Children and Why.* Bristol: Policy Press.

[B31] Gramsci A. (1971). *Selections from the Prison Notebooks.* London: Lawrence and Wishart.

[B32] Hochalf D. Quilter-Pinner H. Kibasi T. (2019). *Ending the Blame Game: The Case for a New Approach to Public Health and Prevention.* Available at: https://www.ippr.org/files/2019-06/public-health-and-prevention-june19.pdf (accessed July 1, 2019).

[B33] Holstein J. Gubrium J. (1997). “Active Interviewing,” in *Qualitative Research: Theory, Method and Practice*, eds Holstein J. Gubrium J. (Thousand Oaks, CA: Sage), 113–129.

[B34] House of Commons Health Committee, (2018). *Childhood obesity: Time for Action.* Available at: https://publications.parliament.uk/pa/cm201719/cmselect/cmhealth/882/882.pdf (accessed September 01, 2018).

[B35] Jones M. Verity F. Warin M. Ratcliffe J. Cobiac L. Swinburn B. (2016). OPALesence: epistemological pluralism in the evaluation of a systems-wide childhood obesity prevention program. *Evaluation* 22 29–48. 10.1177/1356389015623142

[B36] Jones R. Pykett J. Whitehead M. (2013). *Changing Behaviours: On the Rise of the Psychological State.* Gloucestershire: Edward Elgar.

[B37] Kincheloe J. L. McLaren P. (2003). “Rethinking critical theory and qualitative research,” in *The Landscape of Qualitative Research*, second Edn, eds Denzin N. Lincoln Y. (California, CA: Sage), 433–488.

[B38] Lang T. Heaseman M. (2015). *Food Wars: The Global Battle for Mouths, Minds and Markets*, Second Edn Oxon: Earthspan.

[B39] Lau Y. W. Picco L. Pang S. Jeyagurunathan A. Satghare P. Chong S. A. (2017). Stigma resistance and its association with internalised stigma and psychosocial outcomes among psychiatric outpatients. *Psychiatry Res.* 257 72–78. 10.1016/j.psychres.2017.07.027 28734239

[B40] Mahoney C. (2015). *Health, Food and Social Inequality Critical Perspectives on the Supply and Marketing.* Oxford: Routledge.

[B41] Marx K. Engels F. (1845/1998). *The German Ideology.* New York, NY: Prometheus Books.

[B42] McKenzie L. (2012). “The stigmatised and de-valued working class,” in *Class Inequality in Austerity Britain*, eds Atkinson W. Roberts S. Savage M. (Basingstoke: Palgrave Macmillan), 128–144. 10.1057/9781137016386_8

[B43] Miller P. Rose N. (2008). *Governing the Present.* Cambridge: Polity Press.

[B44] Monaghan L. Hollands L. F. Pritchard G. (2010). Obesity epidemic entrepreneurs: types, practices and interests. *Body Soc.* 16 37–71. 10.1177/1357034X10364769

[B45] Murray M. (2015). *Critical Health Psychology.* Basingstoke: Palgrave Macmillan.

[B46] National Obesity Observatory, (2012). *Obesity and the Environment Fast food Outlets.* Available at: http://www.noo.org.uk/uploads/doc/vid_15683_FastFoodOutletMap2.pdf (accessed June 05, 2018).

[B47] Navarro V. (2009). What we mean by social determinants of health. *Glob. Health Promot.* 16 5–16. 10.1177/1757975908100746 19276329

[B48] Nnyanzi L. A. Summerbell C. D. Ells L. Shucksmith J. (2016). Parental response to a letter reporting child overweight measured as part of a routine national programme in England: results from interviews with parents. *BMC Public Health* 16:846. 10.1186/s12889-016-3481-3 27544538 PMC4992560

[B49] O’Hara L. (2014). *The Extent to Which Weight-Related Public Health Initiatives Reflect the Values and Principles of Health Promotion: A Critical Discourse Analysis.* Ph.D. thesis, University of the Sunshine Coast, Sippy Downs, QLD.

[B50] O’Keefe E. (2000). Equity, democracy and globalization. *Crit. Public Health* 10 167–177. 10.1080/713658239

[B51] Ollman B. (2003). *Dance of the dialectic: Steps in Marx’s method.* Chicago, IL: University of Illinois Press.

[B52] O’Malley P. (2008). *Responsibilisation: Sage Dictionary of Policing.* Singapore: Sage.

[B53] Otero G. Pechlaner G. Liberman G. Gurcan E. (2015). The neoliberal diet and inequality in the United States. *Soc. Sci. Med.* 142 47–55. 10.1016/j.socscimed.2015.08.005 26282708

[B54] Parker R. Aggleton P. (2003). HIV and AIDS-related stigma and discrimination: a conceptual framework and implications for action. *Soc. Sci. Med.* 57 13–24. 10.1016/s0277-9536(02)00304-0 12753813

[B55] Peeters R. (2019). Manufacturing responsibility: the governmentality of behavioural power in social policies. *Soc. Policy Soc.* 18 51–65. 10.1017/S147474641700046X

[B56] Pont S. J. Puhl R. Cook S. R. Slusser W. Section On Obesity, and Obesity Society, (2017). Stigma experienced by children and adolescent with obesity. *Pediatrics* 140:e2017303. 10.1542/peds.2017-3034 29158228

[B57] Puhl R. Latner J. D. (2007). Stigma, obesity and the health of the nation’s children. *Psychol. Bull.* 133 557–580. 10.1037/0033-2909.133.4.55 17592956

[B58] QSR International Pty Ltd. (2014). *NVivo Qualitative Data Analysis Software Version 10.*

[B59] Rich E. (2011). “I see her being obesed!”: public pedagogy, reality media and the obesity crisis. *Health* 15 3–21. 10.1177/1363459309358127 21212111

[B60] Ryan G. Bernard H. (2003). *Techniques to Identify Themes.* Thousand Oaks, CA: Sage.

[B61] Saldana J. (2009). *The Coding Manual for Qualitative Researchers.* London: Sage.

[B62] Ramos Salas X. Forhan M. Caulfield T. Sharma A. M. Raine K. (2017). A critical analysis of obesity prevention policies and strategies. *Can. J. Public Health* 108 e598–e608. 10.17269/CJPH.108.6044 31823280 PMC6972457

[B63] Ramos Salas X. Forhan M. Caulfield T. Sharma A. M. Raine K. D. (2019), Addressing internalized weight bias and changing damaged social identities for people living with obesity. *Front. Psychol.* 10.3389/fpsyg.2019.01409 PMC6606721 31293476

[B64] Scambler G. (2018). Heaping blame on shame: ‘weaponising stigma’ for neoliberal times. *Sociol. Rev.* 66 766–782. 10.1177/0038026118778177

[B65] Scambler G. Higgs P. (1999). Stratification, class and health: class relations and health inequalities in high modernity. *Sociology* 33 275–296. 10.1017/s0038038599000176

[B66] Schrecker T. Bambra C. (2015). *How Politics Makes us Sick: Neoliberal Epidemics.* Basingstoke: Palgrave Macmillan.

[B67] Sealy Y. (2010). Parents’ perceptions of food availability: implications for childhood obesity. *Soc. Work Health Care* 49 565–580. 10.1080/00981381003635353 20640967

[B68] Speigelhalter D. Blastland M. (2013). *The Norm Chronicle: Stories and Numbers about Danger.* Basingstoke: Profile Books.

[B69] Steventon E. Sinfield L. Tingle E. Ketteringham C. McKay A. Powell B. (2012). *Childhood Obesity: Parental Feedback and the Weight of Expectation.* Available at: https://www.hsj.co.uk/ (accessed June 05, 2018).

[B70] Sum N. (2012). *Towards a Cultural Political Economy: Discourses, Material Power and (Counter-) Hegemony.* Lancaster: Lancaster University.

[B71] Tronto J. (2013). *Caring Democracy: Markets, Equality, and Justice.* New York, NY: New York University Press.

[B72] Tyler I. Slater T. (2018). Rethinking the sociology of stigma. *Sociol. Rev.* 66 721–743. 10.1177/0038026118777425

[B73] USDA ERS (2013). *New Products.* Washington, DC: Economic Research Service of the US Dept of Agriculture.

[B74] Wainwright M. (2006). The Battle of Rawmarsh. *The Guardian*, 20th September 2006.

[B75] Warin M. Turner K. Moore V. Davies M. (2008). Bodies, mothers and identities: rethinking obesity and the BMI. *Sociol. Health Illness* 30 97–111. 10.1111/j.1467-9566.2007.01029.x 18254835

[B76] Warin M. Zivkovic T. Moore V. Ward P. Jones M. (2015). Short horizons and obesity futures: disjunctures between public health interventions and everyday temporalities. *Soc. Sci. Med.* 128 309–315. 10.1016/j.socscimed.2015.01.026 25645187

[B77] Webb J. Schirato T. Danaher G. (2002). *Understanding Bourdieu.* London: Sage.

[B78] Williams F. (2005). A good-enough life: developing the grounds for a political ethic of care. *Soundings* 30 17–32. 10.3898/136266205820466760

[B79] Winson A. (2014). *The Industrial Diet: The Degradation of Food and the Struggle for Healthy Eating.* New York, NY: New York University Press.

[B80] Zivkovic T. Warin M. Moore V. Ward P. Jones M. (2018). Fat as productive: enactments of fat in an Australian Suburb. *Med. Anthropol.* 37 373–386. 10.1080/01459740.2018.1423563 29319342

