# Supplementary material for: Social Implications of Weight Bias Internalisation: Parents’ Ultimate Responsibility as Consent, Social Division and Resistance
Source: Front Psychol. 2019 Nov 21;10:2321. doi: 10.3389/fpsyg.2019.02321 (PMC6881380; doi:10.3389/fpsyg.2019.02321)

Appendix 1

Phase 1. Topic guide (policymakers and implementers)

Welcome: thanks, first names<br><br>Explanation of project and consent<br><br><u>Screener questions</u><br><br>Just to confirm, you have some involvement in food policy and /or child overweight?<br><br>Information sheet and consent forms – time to read – reschedule?<br><br>Topic guide, show at start

<u>STUDY TITLE: OBESITY DISCONNECT</u>

The policy area is food-related childhood obesity policy. The interview draws on your area of expertise in informing, making or implementing this area of policy.

1. **YOUR WORK AND POLICY**: about your role and involvement around food-related childhood obesity policy

2. **POLICY, SOCIAL GRADIENT AND SOCIAL CLASS**: how does this policy area address the social gradient in obesity in children? How are parents and groups of parents categorised? Is social class relevant?

3. POLICY ASSUMPTIONS AND VALUES: what assumptions or values might there be in policy, if any?

4. **PARENTS INVOLVEMENT WITH POLICY**: to what extent are parents involved in this policy area?

5**. POLICY AND PARENTAL ADVOCATES**: what groups/organisations represent parents' interests?

6. **POLICY INFLUENCERS**: what local groups/organisations have major inputs to this policy area?

7. **CURRENT POLICY AND POLICY OPTIONS**: your views of the strengths, limitations, areas of disconnect, solutions?

Appendix 2

Phase 2. Topic guide (parent participants)

Welcome: thanks, is it ok to use first names?<br><br>The project is about the parents of children with obesity and food policy. I'm interviewing parents (whose children have been classified as obese over the last five years) about their food views, experiences and solutions<br><br><u>Screener questions</u><br><br>Child size: Deprived area: Live/work<br><br>Caregiver age: 18-55<br><br>Child age: 2- 15<br><br>Any reason not to take part:<br><br>Information sheet and consent forms – time to read – reschedule?<br><br>Topic guide, show at start	
	**Question**
	One thing about childhood obesity that is important to you, anything at all
	Shall we use term overweight, obese or??
**1.**	**Thinking about what government says and does are they helping or hindering parents**? (PROMPT materials: C4L, traffic light labels, Disney/other)<br><br>**Why do you say that?**<br><br>**Or** what's government thinking behind this?<br><br>**Thinking about high levels of obesity in children in Lewisham. How do you see the difference in the way government sees the problem and how others see the problem**? **How about you?**<br><br>Prompt how about responsibility? Do any of these matter? SHOW CARDS<br><br>How matter?
**2**	**What impression does government give about parents of overweight children?**<br><br>**What types of family seem to have overweight children?**

**3**	**Higher levels of child overweight in deprived areas…does this make sense to you? What are your thoughts about this?**<br><br>**(if for example says poor) then ask How does this work? Why does this matter or what's that about?**<br><br>If needed say<br><br>**What about social class? Does that matter?**<br><br>PROMPTS – photos shopping areas<br><br>(What differences between wealthier and poorer about food and feeding - what does social class have to do with this)
**4**	**Thinking about how food decisions are made, of the people who advise government who does government take most notice of?**<br><br>**Is government talking to the right people?**<br><br>**Who do you think advises the government on what they should do** (PROMPT CARDS)<br><br>**Are parents involved - how could they be involved**
**5**	**What groups speak up for parents about child health and food**
**6**	**What would you do if prime minister?**<br><br>**(PROMPT CARDS)**<br><br>**Can you see any of these happening? How? What would be needed?**
	Consent forms collect - would like report

Appendix 2 continued

A selection of prompts from the Phase 2 interviews

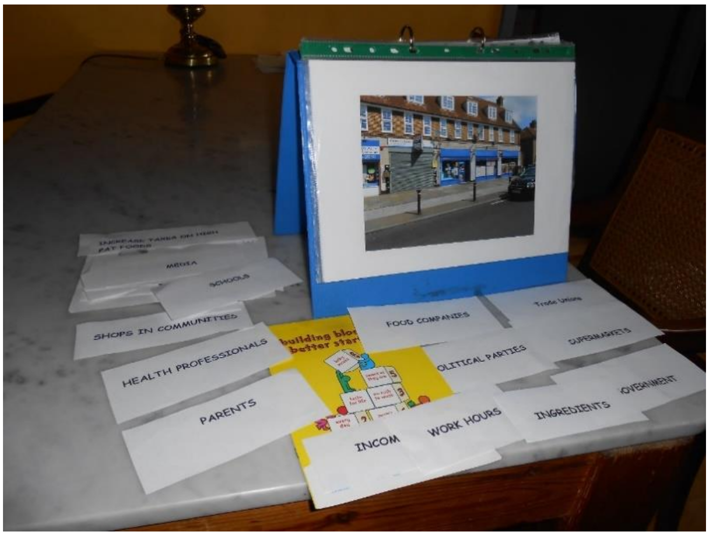

Example of how participant placed cards during interview

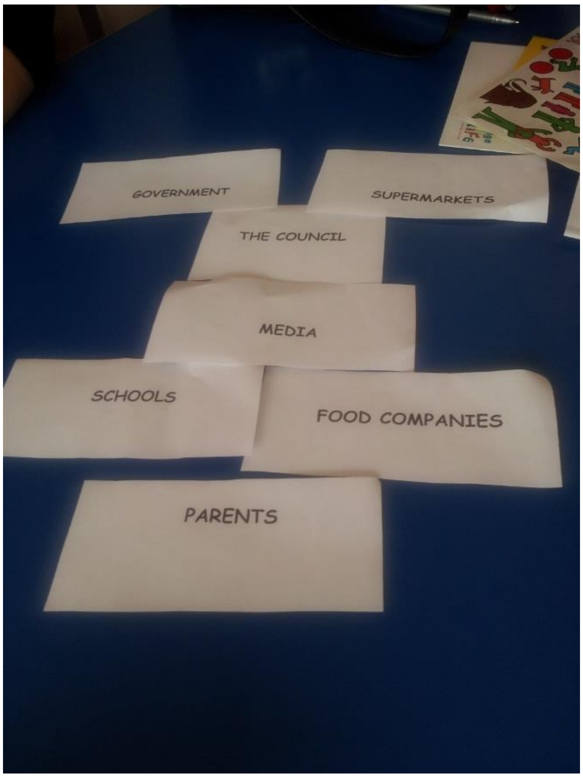

Supplement: Supplementary file 1 [file Data_Sheet_1.pdf]
